# Supplementary material for: 7-Ketocholesterol Promotes Retinal Pigment Epithelium Senescence and Fibrosis of Choroidal Neovascularization via IQGAP1 Phosphorylation-Dependent Signaling
Source: Int J Mol Sci. 2023 Jun 17;24(12):10276. doi: 10.3390/ijms241210276 (PMC10299509; doi:10.3390/ijms241210276)
Supplement: Supplementary file 1 [file ijms-24-10276-s001.zip › ijms-2383083-supplementary.pptx]

## Slide 1
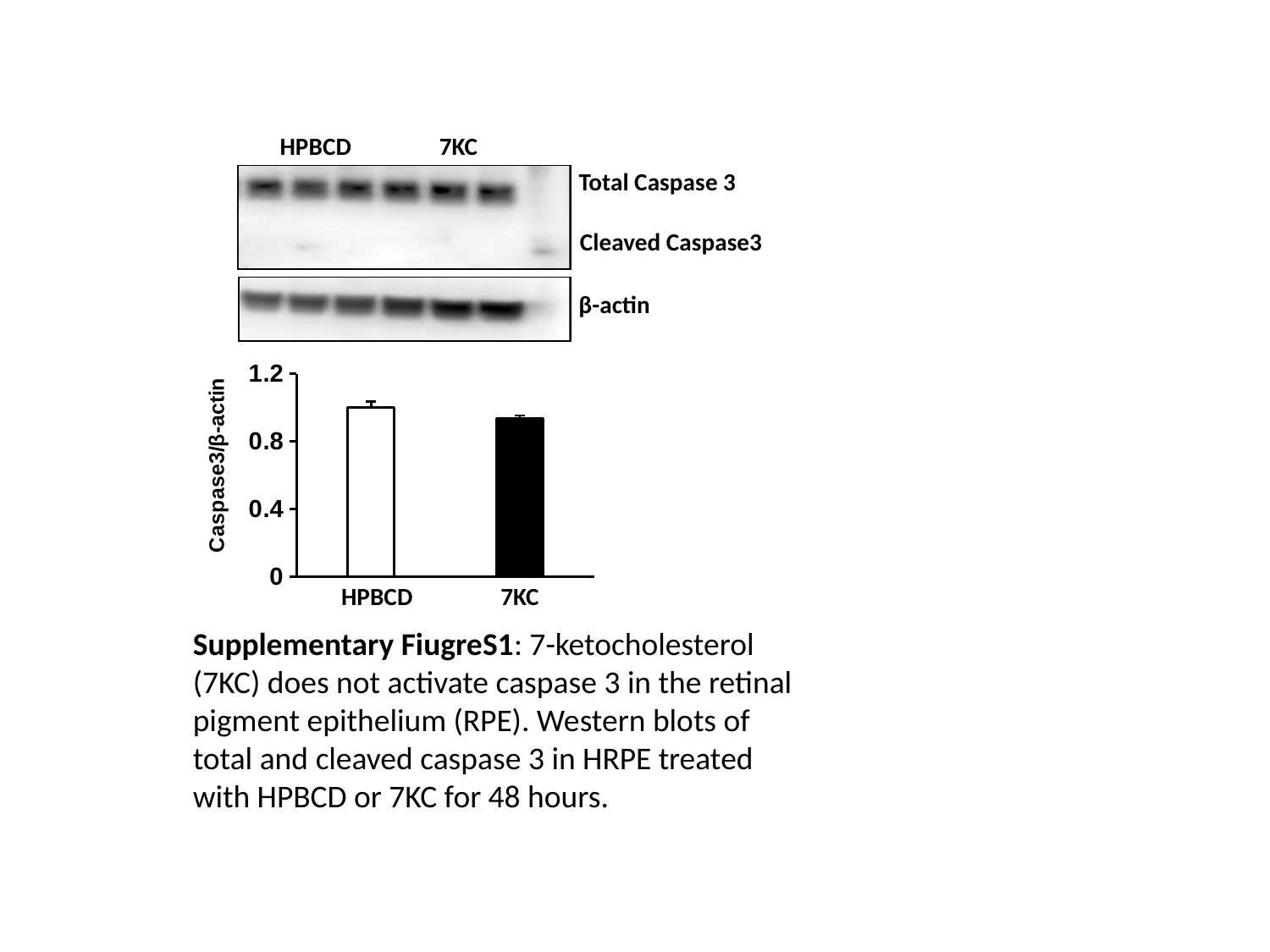

HPBCD 7KC
Total Caspase 3
Cleaved Caspase3
β-actin
### Chart
| Category | |
|---|---|HPBCD 7KC
Supplementary FiugreS1: 7-ketocholesterol (7KC) does not activate caspase 3 in the retinal pigment epithelium (RPE). Western blots of total and cleaved caspase 3 in HRPE treated with HPBCD or 7KC for 48 hours.

## Slide 2
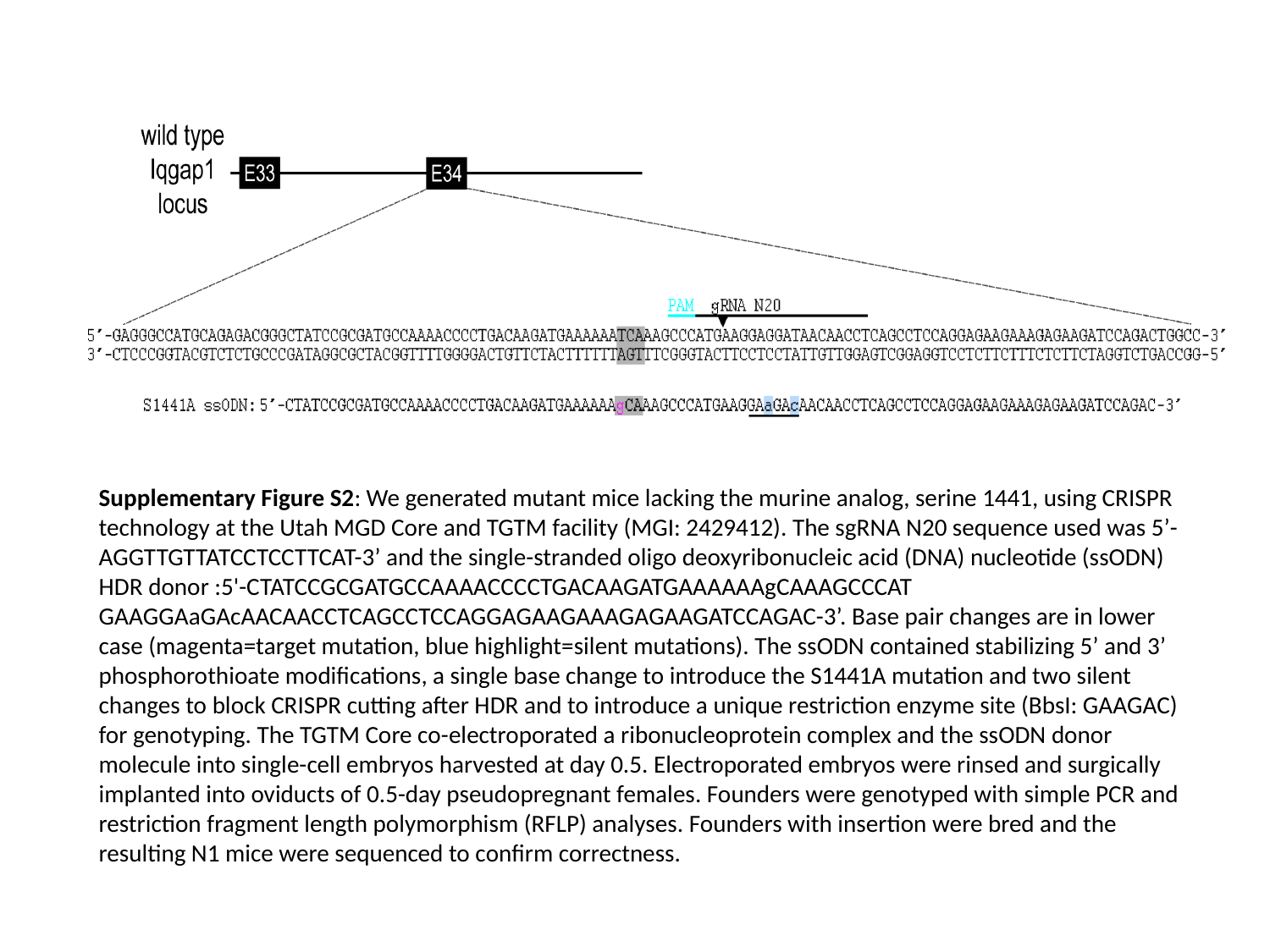

Supplementary Figure S2: We generated mutant mice lacking the murine analog, serine 1441, using CRISPR technology at the Utah MGD Core and TGTM facility (MGI: 2429412). The sgRNA N20 sequence used was 5’- AGGTTGTTATCCTCCTTCAT-3’ and the single-stranded oligo deoxyribonucleic acid (DNA) nucleotide (ssODN) HDR donor :5'-CTATCCGCGATGCCAAAACCCCTGACAAGATGAAAAAAgCAAAGCCCAT GAAGGAaGAcAACAACCTCAGCCTCCAGGAGAAGAAAGAGAAGATCCAGAC-3’. Base pair changes are in lower case (magenta=target mutation, blue highlight=silent mutations). The ssODN contained stabilizing 5’ and 3’ phosphorothioate modifications, a single base change to introduce the S1441A mutation and two silent changes to block CRISPR cutting after HDR and to introduce a unique restriction enzyme site (BbsI: GAAGAC) for genotyping. The TGTM Core co-electroporated a ribonucleoprotein complex and the ssODN donor molecule into single-cell embryos harvested at day 0.5. Electroporated embryos were rinsed and surgically implanted into oviducts of 0.5-day pseudopregnant females. Founders were genotyped with simple PCR and restriction fragment length polymorphism (RFLP) analyses. Founders with insertion were bred and the resulting N1 mice were sequenced to confirm correctness.
